# Gelled Liquid Crystal Nanocarriers for Improved Antioxidant Activity of Resveratrol

**DOI:** 10.3390/gels9110872

**Published:** 2023-11-02

**Authors:** Antonia Mancuso, Martine Tarsitano, Rosy Cavaliere, Massimo Fresta, Maria Chiara Cristiano, Donatella Paolino

**Affiliations:** 1 Department of Experimental and Clinical Medicine, University “Magna Græcia” of Catanzaro, Campus Universitario “S. Venuta”—Building of BioSciences, Viale S. Venuta, Germaneto, 88100 Catanzaro, Italy; antonia.mancuso@unicz.it (A.M.); rosy.cavaliere@studenti.unicz.it (R.C.); 2 Department of Health Sciences, University “Magna Græcia” of Catanzaro, Campus Universitario “S. Venuta”—Building of BioSciences, Viale S. Venuta, Germaneto, 88100 Catanzaro, Italy; martine.tarsitano@studenti.unicz.it (M.T.); fresta@unicz.it (M.F.); 3 Department of Medical and Surgical Sciences, University “Magna Græcia” of Catanzaro, Campus Universitario “S. Venuta”—Building of BioSciences, Viale S. Venuta, Germaneto, 88100 Catanzaro, Italy

**Keywords:** resveratrol, liquid crystals nanocarriers, skin application, antioxidant activity, microrheology, dynamic rheology

## Abstract

As many natural origin antioxidants, resveratrol is characterized by non-suitable physicochemical properties for its topical application. To allow its benefits to manifest on human skin, resveratrol has been entrapped within liquid crystal nanocarriers (LCNs) made up of glyceryl monooleate, a penetration enhancer, and DSPE-PEG 750. The nanosystems have been more deeply characterized by using dynamic light scattering and Turbiscan Lab^®^ Expert optical analyzer, and they have been tested in vitro on NCTC 2544. The improved antioxidant activity of entrapped resveratrol was evaluated on keratinocyte cells as a function of its concentration. Finally, to really propose the resveratrol-loaded LCNs for topical use, the systems were gelled by using two different gelling agents, poloxamer P407 and carboxymethyl cellulose, to improve the contact time between skin and formulation. The rheological features of obtained gels were evaluated using two important methods (microrheology at rest and dynamic rheology), before testing their safety profile on human healthy volunteers. The obtained results showed the ability of LCNs to improve antioxidant activity of RSV and the gelled LCNs showed good rheological profiles. In conclusion, the results confirmed the potentiality of gelled resveratrol-loaded nanosystems for skin disease, mainly related to their antioxidant effects.

## 1. Introduction

The topical application of drugs has emerged as an increasingly favored solution, offering a promising alternative to other administration routes for addressing various medical challenges, particularly when a local effect of a drug is desired. Indeed, a topically applied drug is less involved in causing systemic side effects and it is not subjected to the first-pass effect that should induce a marked reduction in available drug dose [1]. Unfortunately, not all drugs can be effectively administered via the skin route due to their unfavorable physico-chemical features [2].

Thanks to the most modern technologies and discoveries, it has been possible to improve the skin-specific activity of drugs, permitting an efficacious topical administration.

Several nanosystems were designed with the aim to lead the entrapped drug through skin structures, reaching the effective dose at its action sites. Examples include: ethosomes, transfersomes, nanoparticles, and many others which can deliver different natural and synthetic drugs in their structural compartments [3,4,5]. About topical treatment of cutaneous diseases, the numerous successes of nanotechnology reported in the literature demonstrate how important the vehicle is to ensure increased efficacy of drugs that could not be functional if administered as free form to the skin.

Indeed, the vehicle is important to give texture to the formulation, to permit an easy skin application, and also to drive drug penetration through the skin, reaching deeper skin layers if necessary. It is known that the drug can be included in different types of conventional semi-solid formulation, such as pastes, creams, gels, oils, and ointments; however, these examples of conventional dosage form are often not sufficient to improve the drug’s ability to cross the stratum corneum. On the other hand, the application of liquid suspension of nanocarriers could exceed the skin permeation limit but it can be difficult to apply for the patients, and the formulation could drain away leading to the loss of drug and efficacy.

Resveratrol (3,4′,5-trans-trihydroxy-stilbene—RSV) is a natural phenolic compound belonging to the stilbenoid group, obtained from grapes, berries, peanuts, and red wine [6,7]. It gained attention for its potential health benefits including anticancer [8], anti-inflammatory [9], and its antioxidant properties as a function of its concentration and of the duration of the treatment (acute or chronic use) [10].

Like many other natural compounds, RSV has not suitable physico-chemical features that guarantee its passage through the stratum corneum after its skin application. In particular, excessively poor solubility in water severely hampers its permeation ability due to the complex skin barrier structure [11]. Considering the previous assumptions and to overcome the limits of topical application of RSV, the aim of this research work was to provide a new effective topical formulation containing RSV, entrapping it within liquid crystals nanocarriers (LCNs), as promising lipid-based nanosystems which already reported suitable skin penetration properties. For these purposes, LCNs were chosen as the drug delivery systems because of their unique characteristics, high stability, and high compatibility with hydrophilic, lipophilic, and amphiphilic compounds [12,13]. LCNs were prepared using glyceryl monooleate (GMO) as the main component [14] and a mixture of glycerides of oleic acid and other fatty acids that self-assemble when they are in contact with water. As is well known, oleic acid and consequently, GMO, are useful as penetration enhancers, improving the skin permeation of LCNs and of the entrapped drug by promoting the extraction of ceramides and enhancing the lipid fluidity in the stratum corneum [15]. Despite the advantage of LCNs, their use could be limited for topical applications due to low viscosity. To better achieve the aim of the research and to meet the satisfaction of potential patients, the last step of this research work was to jellify the LCNs suspensions by testing different gelling agents, such as poloxamer P407 and carboxymetylcellulose. The rheological properties of the final formulations containing nanosystems have been examined to pinpoint the optimal vehicle for ameliorate the cutaneous applicability of resveratrol, as a potent antioxidant agent. Finally, in vivo tests were carried out to evaluate the safety profile of gelled formulations.

## 2. Results and Discussion

### 2.1. Physico-Chemical Characterization of Empty and RSV-Loaded LCNs

Liquid crystal nanosystems have already been proposed as drug delivery systems for the administration of drugs that do not have suitable features for their use in free-form application [16,17]. In this work we want to evaluate the applicability of gelled modified LCNs for topical administration of Resveratrol, characterized by concentration-dependent antioxidant, anti-inflammatory and anticancer activities being used here as a model drug. Starting from the already investigated formulation [12], we modified the composition including the DSPE-PEG 2000 and DSPE-PEG750 (Table 1). These surfactants, which are components of biological membranes, have been obtained combining a PEG chain and a phospholipid as DSPE [18,19]. The series of DSPE-PEG have already been used in the nanocarriers preparation for the skin target, improving in some cases the skin penetration of entrapped drugs [20]. For this reason, we evaluated the effects of DSPE-PEG 2000 and DSPE-PEG 750 on the physico-chemical features of LCNs. Dynamic light scattering was used as the main technique to characterize the nanosystems from a physico-chemical point of view. In Table 2, the results concerning mean size (nm), polydispersity index (PdI) and zeta-potential (mV) of empty and RSV-loaded LCNs are reported as mean data ± standard deviation.

The evaluation of mean size, polydispersity index and zeta potential is crucial for confirming the applicability of nanosystems for all administration routes, and above all, for topical application. The data in Table 2 shows that the proposed LCN formulations with and without RSV are characterized by suitable physico-chemical features such as mean size included between 70 and 100 nm, polydispersity index values less than 0.3 and a net negative surface charge. These features not only predict real topical applicability but also good stability of the formulations. In particular, the shown zeta potential values fair from neutrality confirm the repulsion forces established between similarly charged nanosystems, preventing instability phenomena such as creaming, sedimentation or flocculation [21].

**Figure 1:**
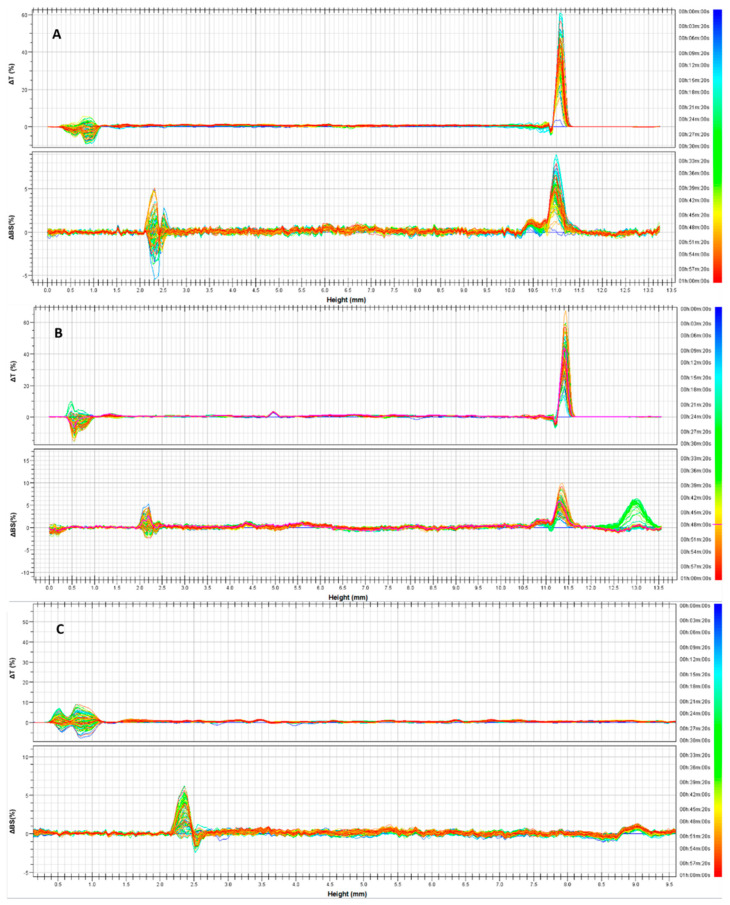
Delta transmission (ΔT) and delta backscattering (ΔBS) profiles as a function of time and sample height (mm) obtained by using a Turbiscan Lab^®^ Expert for empty formulations: (**A**) LCN-A, (**B**) LCN-B, and (**C**) LCN-C. The analysis was carried out for 1 h at room temperature. The reported data are representative of three different analyses performed on three batches of each formulation.

This hypothesized stability was confirmed by a Turbiscan Lab^®^ Expert analysis. In Figure 1, delta transmission and delta backscattering profiles of the three empty formulations are reported as a function of analysis time (1 h) and the sample’s height (8 mm). As can be seen, the variation of each profile was within values of ±5%, for the entire height of the samples; thus indicating that no instability phenomena occurred. In fact, each profile has a different color and represents a scan of each sample at a different time (within 1 h of analysis). As can be seen, the various scans are superimposable. The alterations of profiles recorded below 2 mm and above 8 mm of sample height should not be considered because they are caused by the presence of air bubbles at the bottom of the vial and at the liquid–air interface, respectively [22].

Observing Table 2, it is possible to note that the presence of increasing concentration of RSV within nanosystems does not negatively influence the physico-chemical values in comparison with empty formulations. Moreover, the addition of the DSPE-PEG 2000 and DSPE-PEG750, respectively, in the LCN-B and LCN-C formulations induced a slight reduction in mean size if compared to LCN-A. This trend could be due to the surfactants added during the preparation phase resulting in more malleable systems. The entrapment efficiency is another important parameter to be evaluated for proposing drug delivery systems. So, in our study promising results were obtained in terms of drug retention and loading capacity. In detail, each pool of formulation reported an increase in loading capacity as a function of the increase in the drug dose used for sample preparation. The formulations LCN-A, characterized by its larger dimensions, are also the best to contain the drug. Probably the absence of surfactant induces the formation of more rigid structures capable of retaining the lipophilic RSV inside them.

**Figure 2:**
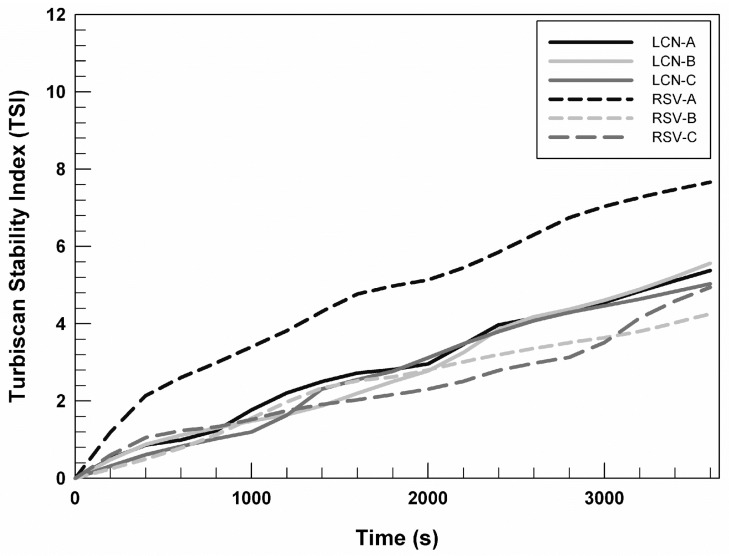
Turbiscan stability index (TSI) of different formulations (empty LCN and RSV (0.72 mg/mL)-loaded LCNs) obtained by using a Turbiscan Lab^®^ Expert as a function of time (1 h). The analyses were performed at room temperature and the reported data are representative of three different analyses carried out on three batches of each formulation.

In Figure 2, we reported the Turbiscan stability index (TSI) profiles of RSV (0.72 mg/mL)-loaded LCNs and empty nanosystems as a function of time (1 h), to compare their stability over time. It is possible to note that profiles relating to empty formulations are almost superimposable. However, the TSI profile of RSV-A prepared by using 0.72 mg/mL of drug differs from the profiles of other formulations, suggesting that the presence of the chosen drug induces a slight but not relevant destabilization of the system. Whereas, the stability profiles of RSV-B and RSV-C, at the RSV concentration equal to 0.72 mg/mL, are superimposable on the profiles of the respective empty formulations. This trend permitted us to hypothesize that the presence of DSPE-PEG 2000 and DSPE-PEG 750 in LCN-B and LCN-C can induce a stabilization of the system even in the presence of drugs.

The TSI profiles of RSV-loaded LCNs prepared using 0.24 and 0.48 mg/mL are superimposable on RSV (0.72 mg/mL)-loaded LCNs, for this reason we chose to show the stability trend of LCNs prepared with the highest RSV concentrations.

### 2.2. In Vitro Evaluation of LCNs Safety Profile

**Figure 3:**
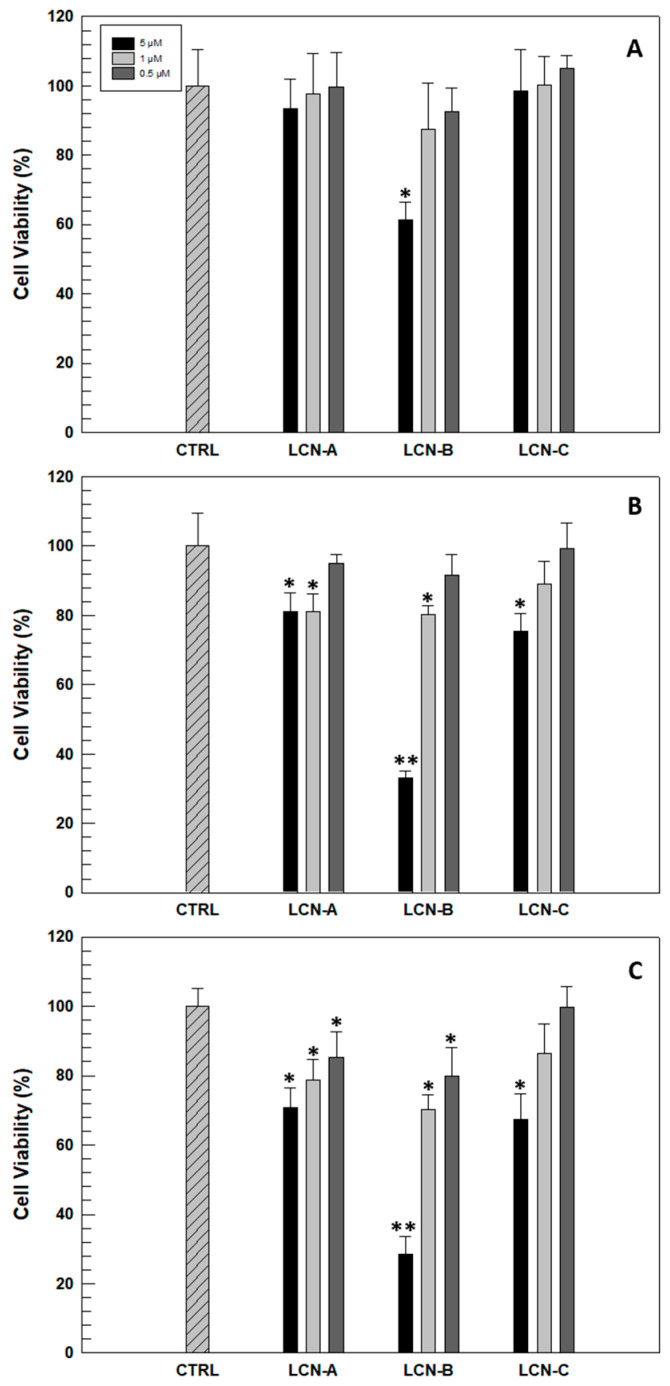
Cytotoxic effect of empty LCNs on human NCTC 2544 cell-line as a function of time ((**A**) 24 h, (**B**) 48 h, and (**C**) 72 h) and the amount of nanosystems used to reach effective concentrations of RSV in the subsequent in vitro experiments (5, 1 and 0.5 µM). For each experiment, carried out by MTT test, untreated cells have been used as a control (CTRL). Results are expressed as the average of five different experiments ± standard deviation. * *p* ≤ 0.05; ** *p* ≤ 0.001.

Before evaluating the antioxidant efficacy of RSV-loaded LCNs on a human cell model, an in vitro evaluation of safety profile is necessary. This was investigated in human keratinocyte NCTC 2544 cells selected as an in vitro model. The possible cytotoxicity was evaluated as a function of incubation time and the amount of LCNs necessary to reach the effective and chosen RSV concentrations (5, 1 and 0.5 µM). As can be seen in Figure 3, only LCN-B formulation induced a significant reduction in cell viability after 24 h of treatment and at the highest concentration. This result was expected since formulation B previously showed the lowest drug retention capacity, and consequently, larger quantities of nanosystems are needed to reach the RSV concentration of 5 µM. The reduction of NCTC 2544 cells viability becomes accentuated in the following 48 and 72 h when the cells have been treated with the highest concentration of LCN-B. However, LCN-C formulation showed the best safety profile, leading to a significant reduction in cell viability after 72 h of treatment and at the highest concentrations.

Considering these in vitro results, LCN-B formulation was excluded from subsequent studies.

### 2.3. In Vitro Evaluation of Protective Effects of RSV-Loaded LCNs on NCTC 2544 Cells Exposed to Oxidant Agent

Oxidative stress, caused by different potential agents such as hydrogen peroxide or UV radiation, is the basis of skin cellular injuries which lead to various pathological conditions such as skin-aging and cancer [23]. Components of natural origin, a resveratrol, have widely demonstrated protective effects against oxidant. Unfortunately, resveratrol as free-form resveratrol is not suitable for eliciting its antioxidant effects on human skin due to its high lipophilicity that makes it not suitable for topical application [24]. To deliver and to improve in vitro protective effects of RSV, the drug was entrapped within different LCNs, and on the basis of in vitro results described in Section 2.2, the formulations LCN-A and LCN-C have been chosen for testing the protective effects of entrapped RSV on human keratinocytes in terms of cell viability and LDH release after oxidative stress.

In in vitro studies we tested the formulations A and C prepared with 0.72 mg/mL of RSV, since they have shown the best physico-chemical features above all in terms of entrapment efficiency and stability. In detail, to evaluate the protective effects of RSV and RSV-loaded LCNs, keratinocytes were treated for 24 h with 0.5 µM and 1 µM of drug as free and encapsulated form. At the end of treatments, cells were exposed to H_2_O_2_ (700 µM) for 1.5 h to induce the oxidant process and damage to cell membranes [25].

In this case, cell viability evaluation and the quantification of released LDH (lactic hydrogenase) are methods that permit to evaluate indirectly the effects of treatments on cells exposed to oxidizing agents. When cells are treated with pro-oxidant factors, they suffer damage to the cellular membrane with an increase in its permeability; the consequences of this damage are generally an increased release of LDH and in many cases the death of cells.

**Figure 4:**
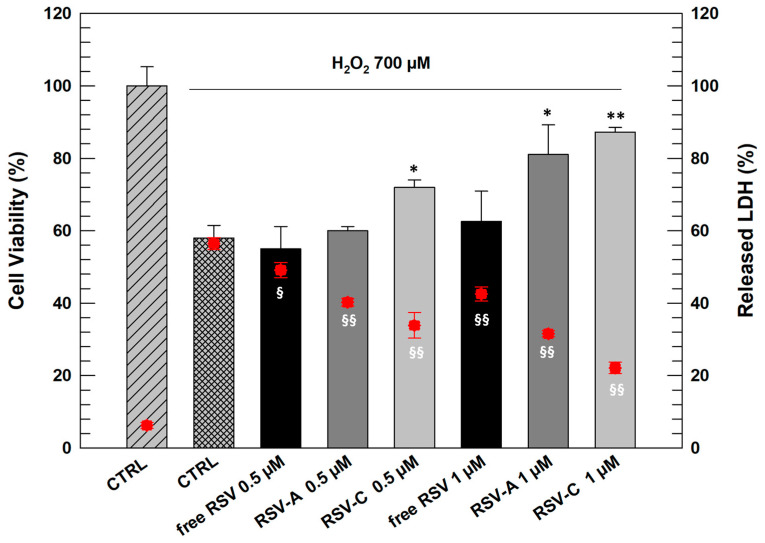
In vitro evaluation of protective effects of free RSV and RSV-loaded LCNs carried out by MTT test (grey bars) and LDH assay (red plots) on NCTC 2544. For each experiment, untreated cells have been used as a control (CTRL). Results are expressed as the average of five different experiments ± standard deviation. * *p* < 0.05 and ** *p* < 0.001 for cell viability versus CTRL treated with H_2_O_2_; § *p* < 0.05 and §§ *p* < 0.001 for released LDH, versus CTRL treated with H_2_O_2_.

As can be seen in Figure 4, NCTC 2544 cells responded to oxidative stress when treated with H_2_O_2_ (700 µM) with a significant reduction of cell viability (42% of mortality) and a significant increase in LDH release (56.45% ± 1.65 respect to 6.25% ± 0.85 of untreated cells).

RSV as free-form at both tested concentrations proved incapable of carrying out a significant protective action on cells after exposure to H_2_O_2._ However, the cells pre-treated with both chosen LCNs (A and C) resulted in more protection from oxidative stress. In detail, formulation RSV-C already at lowest concentration (0.5 µM) induced a significant increase in NCTC 2544 viability and reduction in LDH release with respect to positive control. RSV-A at 0.5 µM had beneficial effects on cells but not as marked and significant as formulation C. This result can be justified by the presence of DSPE-PEG 750 in the composition of LCN-C, that probably make them more flexible and more able to interact with cell membrane inducing intracellular release of entrapped RSV [26].

Increasing the RSV concentration entrapped within LCNs, we obtained significant results also with LCN-A since this formulation showed to be able increasing cell viability of stressed cells (81.03% ± 8.2) significantly reducing the release of LDH from cells (31.56% ± 0.85).

### 2.4. Rheological Characterization of Gelled LCNs

The results shown so far have demonstrated that LCNs, and in particular the formulation A and C, are suitable for potential skin delivery of RSV. However, a potential topical application of these nanosystems requires the design of an easier and more comfortable dosage form. Indeed LCNs, such as other topical nanosystems, i.e., transfersomes, ethosomes, and niosomes, are characterized by very low viscosity, similar to water, and their application on the skin could be difficult and lead to their slipping away from the application site. Starting from these assumptions, we included LCNs within three-dimensional networks made up of carboxymethylcellulose (CMC) or poloxamer P407 (P407), with the aim to increase the contact time between skin and nanosystems. The choice fell on these two materials due to their gelling properties, ready availability, and low cost.

For this study, LCN-A and LCN-C were gelled (LCN-B formulation was excluded after in vitro studies) using P407 and CMC, reaching a concentration equal to 20% and 5%, respectively. In our previous study we demonstrated that the percentage of 20% for P407 is the best compromise between functionality and costs [27]. On the other hand, Ghannam and Esmail demonstrated that CMC-based gel was characterized by non-Newtonian rheological behavior that is important for spreadability [28,29,30].

The obtained gelled LCNs were analyzed by using two different tools: the Rheolaser Master™ and Kinexus Pro+ rotational rheometer, obtaining important information about the rheological behavior of samples at rest and under solicitation, respectively.

#### 2.4.1. Microrheological Investigation of Gelled LCN-A and Gelled LCN-C

For microrheological characterization, LCN-A and LCN-C were gelled by using carboxymethyl cellulose (5% *w*/*w*) and poloxamer P407 (20% *w*/*w*). The Rheolaser Master™ analysis is based on diffusing wave spectroscopy and on the Brownian movement of particles contained in the sample; this is a technique that allows the non-invasive evaluation of a material’s rheological properties [31]. The movement of particles can cause the deformation of speckle images and a specific detector records the entity of this deformation, quantifying the velocity of particle movement [32]. The freedom of movement of the particles is certainly dependent on the elasticity and viscosity of the matrix in which they find themselves. In a “soft gel” or fluid, the particles are in a structure that does not hinder their movement. In this case, the slope of MSD (mean square displacement) curves as a function of decorrelation time is directly proportional to the medium viscosity. However, when particles are included in a “hard gel”, a more structured and visco-elastic gel, their movement is strongly limited. Thus the slope of MSD curves as a function of decorrelation time decreases until a plateau is reached [33]. Since the decorrelation time can be compared to frequency in a traditional rheological analysis, the MSD curves represent the viscoelastic behavior of a three-dimensional network, as hydrogel, in a frequency domain [31]. Mean square displacement is a fundamental microrheological parameter because it is totally based on the diffusive Brownian motion thanks to which, the particles do not move in specific direction but randomly move within the available space. Thus, the displacement is measured as a squared distance (nm^2^) [34].

**Figure 5:**
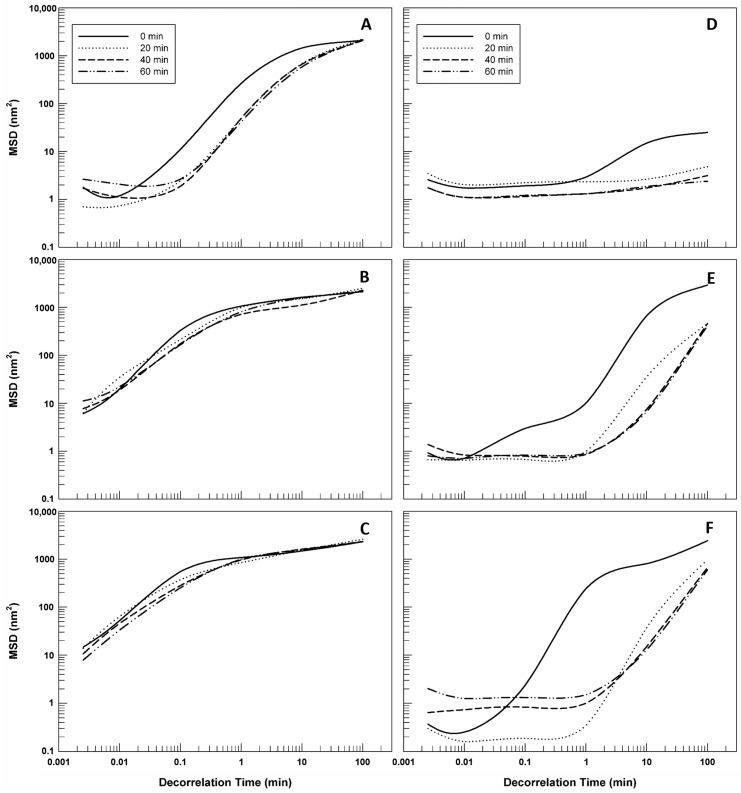
Mean square displacement MSD of gelled samples as a function of decorrelation time. The illustrated results were representative of three independent experiments. Legend: (**A**) 5% CMC-based gel; (**B**) 5% CMC-based gel with LCN-A; (**C**) 5% CMC-based gel with LCN-C; (**D**) 20% P407-based gel; (**E**) 20% P407-based gel with LCN-A; (**F**) 20% P407-based gel with LCN-C.

Figure 5 reports the MSD curves of all gelled samples and comparing them it is immediately possible to notice the different behavior between CMC- and P407-based gels. The mean square displacement curves as a function of decorrelation time related to CMC-based gels (panel A) are greater than MSD curves of P407-based gel (panel D); namely, the MSD curves of carboxymethyl cellulose gel reached maximum values of ~2000 nm^2^, while the MSD curves of P407-based gels remained under values of ~25 nm^2^. This marked difference between data reported in panel A and panel D of Figure 5 highlights the different behavior from the rest of the samples. CMC-based gel was found to be less viscous than poloxamer gel and hence the particles inside move more freely [35]. The presence of LCN-A and LCN-C in CMC-based gels does not affect the trend of MSD curves, confirming that at rest the LCNs do not alter the mesh and the microrheological behavior of carboxymethyl cellulose gels. However, LCN-A (Figure 5E) and LCN-C (Figure 5F) led to an increase in MSD curve slope at high decorrelation time in P407-based samples, maintaining in any case lower than CMC-based gels.

Processing the MSD data, by using RheoSoft Master^®^ 1.4.0.0 software, other microrheological parameters can be obtained: elasticity index (EI) and solid liquid balance (SLB).

**Figure 6:**
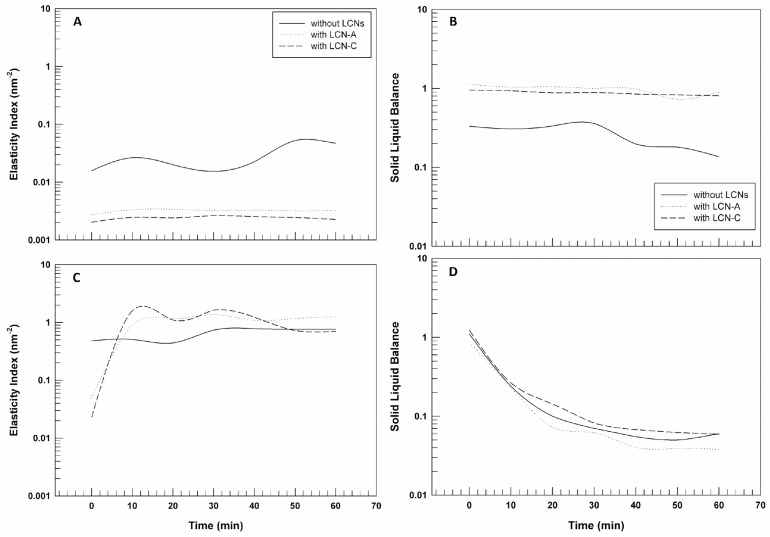
Elasticity Index (EI) and Solid Liquid Balance (SLB) curves of 5% CMC-based gels (panel (**A**,**B**), respectively) and 20% P407-based gels (panel (**C**,**D**), respectively) as a function of time. The illustrated results were representative of three independent experiments.

The EI curves reported in Figure 6 panels A and C confirm what has already been shown, considering that the elasticity index corresponds to the inverse of particles’ movement velocity within the gelled structure [36]. The elasticity index values of CMC-based gels with and without LCNs are significantly lower than those of poloxamer gel, thus demonstrating the different movement freedom of the particles of the two pools of samples. Carboxymethyl cellulose seems to lead to the formation of a network less rigid and structured and hence particles seem to move more freely.

Finally, the solid–liquid balance (SLB) describes the ratio between the liquid-like and solid-like behavior of the samples. The SLB = 0.5 represents that the liquid and solid parts are equal. A value of 0 < SLB < 0.5 means that the solid behavior dominates, and the gel network is considered more elastic or solid-like; however, a value of 0.5 < SLB < 1 indicates that the liquid behavior is predominant [34]. Figure 6 panel B highlights that the gels prepared by using CMC have a predominant liquid-like behavior, which does not mean that the samples are liquids, but that their structure is more malleable and softer. The SLB values related to P407-based gels are very close to 0.1, confirming the predominance of solid-like behavior and a more structured three-dimensional network in which the particles’ movements are limited.

#### 2.4.2. Dynamic Rheological Characterization of Gelled LCNs

The microrheological analysis of samples is important since it permits to characterize samples at rest, without modifying their structures and observing their inherent rheological behavior. But when a formulation is designed to be topically applied and spread onto the skin, the analysis of its rheological behavior under solicitation becomes fundamental. For this reason, viscosity measurements for evaluating the sliding properties, and oscillatory measurements for evaluating the deformability properties of the gels were carried out. Through these analyses, parameters such as dynamic viscosity, G′ and G″ have been obtained.

**Figure 7:**
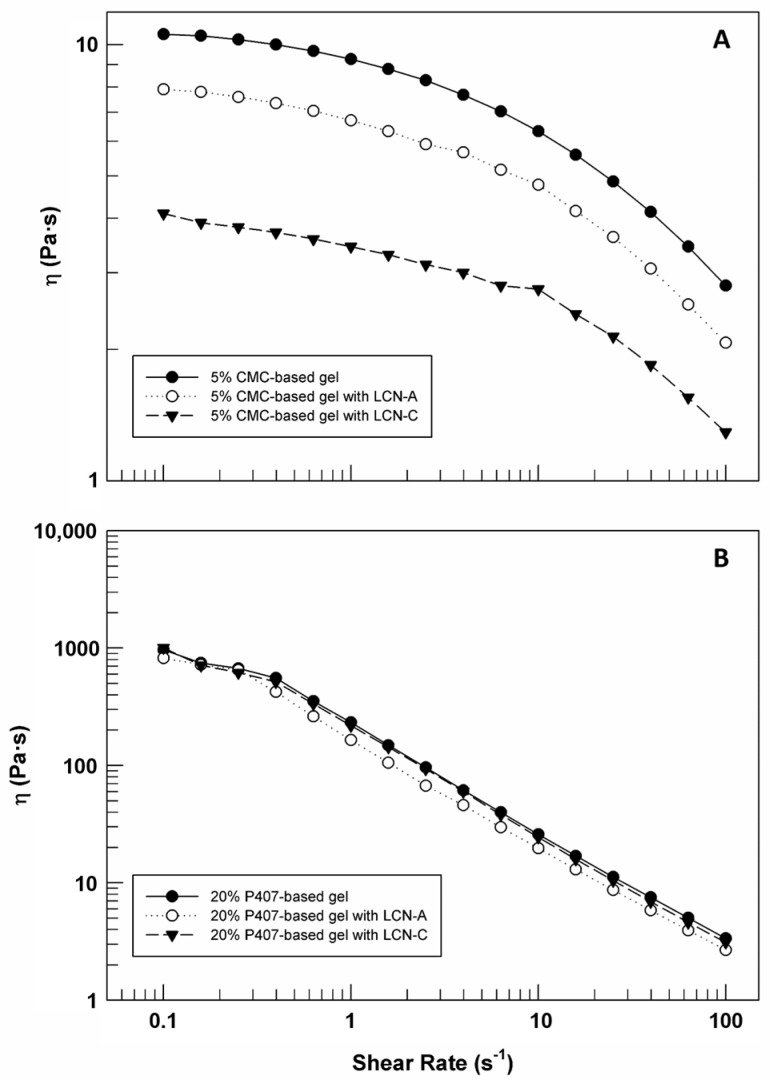
Flow curves (shear viscosity versus shear rate) of 5% CMC-based gel (**A**) and 20% P407-based gel (**B**), with and without carriers. The illustrated results were representative of three independent experiments.

Figure 7 shows the flow curves of CMC-based gels (panel A) and P407-based gels (panel B) prepared with and without LCNs. As can be seen in the figure, all samples showed a non-Newtonian behavior since their viscosity values decreased when shear rate was increased. This means that the CMC and P407 gels can modify their inner structure and flow when the solicitation is applied.

In detail, for CMC-based gels (Figure 7A) the reduction of viscosity is not sudden, but rather gradual, both in presence and in absence of LCNs. Moreover, it is easy to note that the presence of LCN-A and LCN-C induced a reduction in starting viscosity and a modification of curves slope. The effect of LCN-C on flow curves is more evident than other LCN formulations. It could be due to the presence in the formulation C of unreacted DSPE-PEG 750 molecules that could induce their fluidizing effects on the mesh of a three-dimensional network made up of CMC. In Table 3 the average values of shear viscosity (±standard deviation) were reported for all samples as a function of different shear-rate values.

Comparing the data reported in Figure 7 and in Table 3, the different response to the same solicitation of CMC-based gels and P407-based gels is evident. The starting viscosity of P407-based gels is about 100-times higher than CMC-based gels viscosity, regardless of LCNs, as already demonstrated by MSD curves (Figure 5). Moreover, the flow curves obtained from the rheological analysis of P407-based gels, with and without LCN-A and LCN-C (Figure 7B), are almost overlapping; demonstrating that LCNs do not disturb the structure and rheological behavior of the three-dimensional network formed via poloxamer P407. A higher viscosity of gels could be exploited to retain the formulation longer at the application site of skin [29].

The slope of flow curves related to P407-based gels is higher than the slope of viscosity curves related to other pool of formulations, highlighting that poloxamer gels respond better and more quickly to the applied stress (shear rate) by modifying their internal structure and therefore inducing their own flow. So, it is possible to affirm that despite the viscosity of P407-based gels being higher than CMC-based gels, the first ones are characterized by higher spreadability, since this aspect is directly connected to the reduction in viscosity when a solicitation is applied [29]. This property is not affected by LCNs within the three-dimensional network of poloxamer.

**Figure 8:**
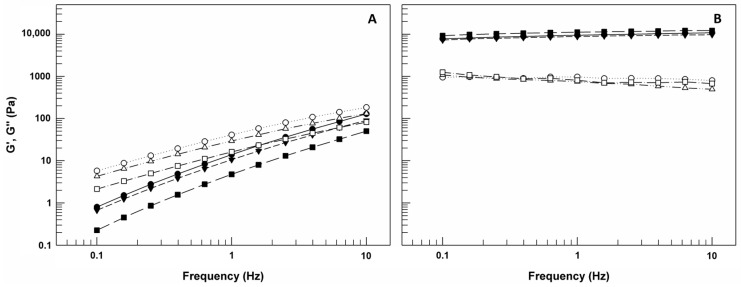
G′ and G″ moduli (Pa) curves versus frequency (Hz) for CMC-based gels (**A**) and P407-based gels (**B**), with and without LCNs. The illustrated results were representative of three independent experiments. Legend panel A: full and empty circles = Gwithout LCNs. The illustrated and G″ moduli of 5% CMC-based gel, respectively; full and empty triangles = G′ and G″ moduli of 5% CMC-based gel with LCN-A, respectively; full and empty squares = G′ and G″ moduli of 5% CMC-based gel with LCN-C. Legend panel B: full and empty circles = Gwithout LCNs. The illustrated and G″ moduli of 20% P407-based gel, respectively; full and empty triangles = Gwithout LCNs. The illustrated and G″ moduli of 20% P407-based gel with LCN-A, respectively; full and empty squares = Gwithout LCNs. The illustrated and G″ moduli of 20% P407-based gel with LCN-C.

The oscillation test carried out by frequency sweep measurements permitted to obtain values about G′ and G″ moduli as a function of frequency (Hz) (Figure 8). G′, also called as storage modulus, is indicative of the stress energy temporarily stored in shear and when G′ is high means that the material can recover its shape and structure after the force; G″ instead represents the energy used for flow and it is also called as loss modulus since this energy is an irreversible loss [37]. The ratio between these two rheological parameters gives an idea on the behavior of analyzed samples. In detail, if G′ is greater than G″, the gel is more similar to solid sample; however, if G″ is greater than G′, the sample shows a liquid-like behavior [38].

As shown in Figure 8A, G″ modulus values of CMC-based gels are greater than G′ values for the entire frequency range, and this trend occurs also in presence of LCN-A and LCN-C. This domain of G″ on G′ indicates that the viscous component of samples may eventually be greater than the elasticity under the continuous high frequency [37]. In the case of CMC-based gels, as already confirmed by flow curves, the presence of LCNs within matrices induced a significant reduction of both G′ and G″ values.

However, observing the panel B of Figure 8, we can note that the G’ curves and G″ curves for P407-based gels remain almost parallel without ever intersecting in the frequency range used and G′ values are greater than G″ for the entire frequency range, regardless of LCNs. These results confirm the prevalence of solid-like behavior of P407-based gels and their ability to resist stress without converting its structure from gel to sol. This is important because in case of gel–sol transformation as response to solicitation, a reduction in contact time between formulation and skin could occur.

### 2.5. In Vitro Permeation Studies through Human SCE Membranes

As already mentioned, RSV has not suitable features for crossing stratum corneum as free-form since a certain balance between lipophilicity and hydrophilicity of drug(s) is required for partition in stratum corneum and into the viable epidermis [39]. In the case of RSV (logP = 3) or other similar drugs, the help of drug delivery systems is necessary to overcome the human barrier and reach action sites [40]. For evaluating the ability of LCNs to interact with human skin and to induce the release of entrapped RSV, percutaneous permeation studies through human skin were carried out. In this phase of study, the ability of LCNs and gelled LCNs to induce the permeation of RSV was compared with RSV applied as free-form on human stratum corneum.

**Figure 9:**
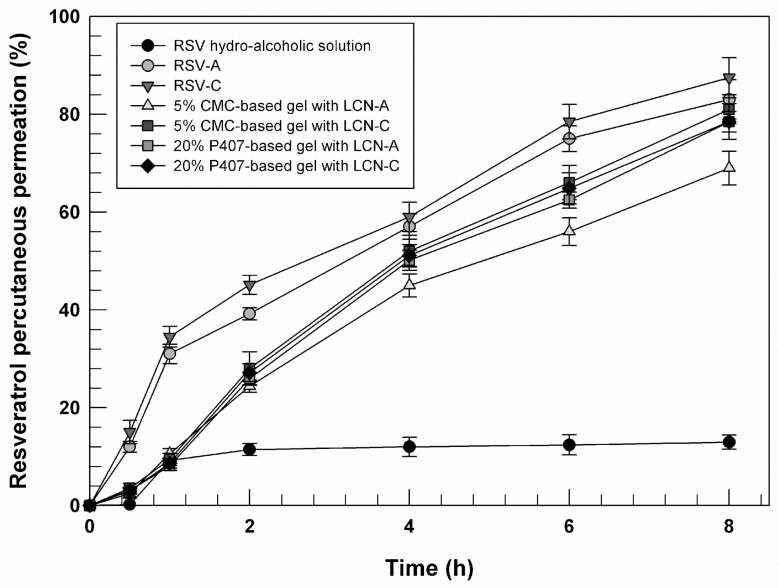
In vitro percutaneous permeation profiles of RSV from LCNs and gelled LCNs compared to RSV applied as free form (hydro-alcoholic solution). The experiments were carried out through SCE membranes and by using Franz diffusion cells. Values represent the mean of three different experiments ± standard deviation.

The most evident trend reported in Figure 9 is the permeation profile of RSV as free form. The drug, probably due to its high lipophilicity, was poorly detected in the receptor medium, confirming its predictably poor ability to cross the stratum corneum and permitting us to hypothesize its accumulation in the stratum corneum [40]. In detail, only 12.97% ±1.45 of applied free RSV in the donor compartment of Franz cells was permeated through the stratum corneum, showing values significantly lower than other formulations (*p* < 0.001). On the other hand, the ability of the proposed formulations in increasing the percutaneous permeation of RSV has also been confirmed. As we can see from Figure 9, all formulations (LCNs and gelled LCNs) are able to induce a permeation of drug greater than 40% of the dose applied after 4 h of experiment. Moreover, the non-gelled LCNs seem more effective compared to gelled formulation. The main component of LCNs is a GMO that is well-known as a permeation enhancer and is able to improve the skin permeation of LCNs and enhance the lipid fluidity in the stratum corneum [41]. When RSV-loaded LCNs are used in gel form, a reduction in RSV permeation is recorded. Probably the formation of a three-dimensional network around nanosystems limited the interaction between LCNs and skin lipids, but without their effectiveness when compared with the hydro-alcoholic solution of RSV.

### 2.6. In Vivo Evaluation of Safety Profile and Gelled LCNs Effects on Skin

Despite a prolonged occlusion of skin which could induce damage to the stratum corneum, the occlusion is normally used in clinical dermatology to induce the transcutaneous penetration of topically applied drugs [42,43]. For this reason and considering that the proposed formulations were designed for the topical delivery of resveratrol, in vivo studies were carried out in occlusive condition on healthy human volunteers. Before starting the study, baseline TEWL values for the six tested cutaneous sites were recorded and no significant differences were found. For the study, saline solution (NaCl 0.9% *w*/*v*) and empty occlusive patches were used as negative and positive controls for occlusion, respectively. Transepidermal Water Loss (TEWL) is used as an indicator of the integrity of the skin. High TEWL values indicate a probable disruption of the skin barrier and a consequent loss of high amounts of physiological cutaneous water with drying out of skin [44].

**Figure 10:**
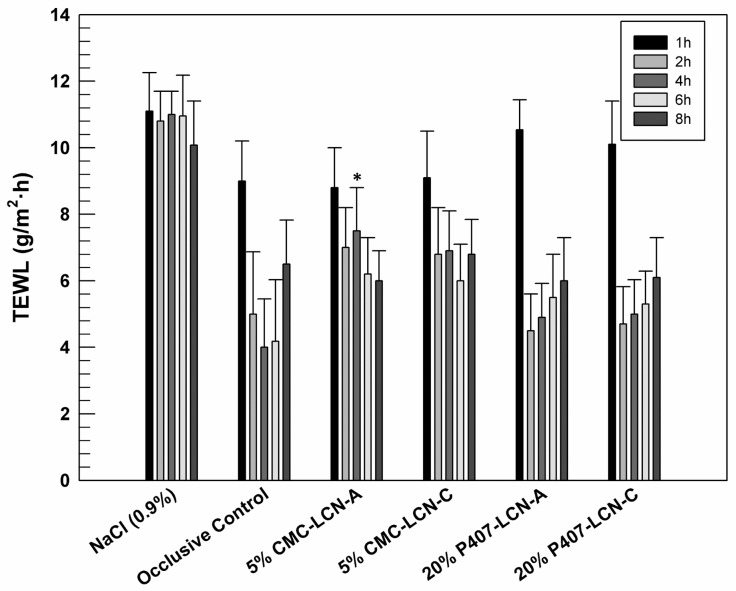
Evaluation of safety profile of gelled LCNs by recording TEWL values over time, after the application of samples on healthy human volunteers (*n* = 10, totally) in occlusive conditions. Results are expressed as the mean values ± standard deviation. * *p* < 0.05 respect to occlusive control at the same time.

The graph reported in Figure 10 shows a significant reduction (*p* < 0.001) in TEWL values related to occlusive control after 2 h of experiments obtained from topically administered saline solution by using the commercial occlusive patch. The occlusion temporarily prevents the epidermal water flux [45].

The applied gelled LCNs generally do not induce detectable damage to the stratum corneum since the TEWL values remained below the values related to the control saline solution. After 2, 4 and 6 h of experiments, all tested formulations induced an increase in TEWL values if compared to occlusive control at the same times. Probably, this effect could be due to the presence of aqueous material within used patches attributable to gels that tend to evaporate when patch is removed for analysis. This hypothesis is confirmed by the TEWL values detected after 8 h of experiments, when no traces of formulations were visible on the volunteer’s skin and TEWL tends to restore.

## 3. Conclusions

The results shown in this research highlight the suitability of liquid crystal nanocarriers being used for improving the percutaneous permeation and the antioxidant activity of resveratrol, a natural multi-beneficial drug. Among three different formulations, we have chosen LCN-A and LCN-C made up respectively of only GMO and GMO with DSPE-PEG 750. The presence of PEG derivatives in the second formulation seems to influence on antioxidant activity of resveratrol-loaded LCN-C and the ability of nanosystems to retain suitable amounts of drug. In the second part of this study, the chosen LCNs were gelled by using two different gelling agents, i.e., poloxamer P407 and carboxymethyl cellulose. The rheological characterization of gelled LCNs showed the sensitivity of some rheological parameters of CMC-based in presence of LCNs, that induces a reduction in flow curves. However, P407-based gels maintained their profiles also with nanosystems. Flow-curves slope and microrheological parameters (EI and SLB) have not undergone significant changes, demonstrating that the gelled systems can be easily spread and used for topical application. Finally, in vivo studies carried out on healthy human volunteers showed that gelled LCNs do not alter in occlusive condition the integrity of stratum corneum, preserving the transepidermal water loss. In summary, our research convincingly demonstrates that the use of nanoscale carriers in gels represents a promising approach to improve the efficacy of numerous therapeutic treatments, opening new perspectives for innovation in drug delivery and biomedical applications.

## 4. Materials and Methods

### 4.1. Materials

Glyceryl monooleate (GMO—Monomuls 90-O18) with purity >90%, 3-(4,5-dimethylthiazol-2-yl)-3,5-diphenyltetrazolium bromide salt (used for MTT-tests), RSV (resveratrol), phosphate buffered saline (PBS) tablets, dimethyl sulfoxide, amphothericin B solution (250 μg/mL), CMC (medium viscosity carboxymethylcellulose), DSPE-PEG-2000 (1,2-distearoyl-sn-glycero-3-phosphoethanolamine-N-[methoxy(polyethylene glycol)-2000]) and DSPE-PEG-750 (1,2-distearoyl-sn-glycero-3-phosphoethanolamine-N-[methoxy(polyethylene glycol)-750]) were purchased from Sigma–Aldrich (Milan, Italy). Poloxamer P407 (Pluronic F127, PL F127), was purchased from BASF (Ludwigshafen, Germany). Ethanol was obtained from Carlo Erba SpA (Rodano, Italy), while cellulose membrane Spectra/Por MWCO 10 kDA was obtained from Spectrum Laboratories Inc. (Eindhoven, The Netherlands).

For the in vitro studies, NCTC2544 cells were provided by the Instituto Zooprofilattico of Modena and Reggio Emilia (Instituto Zooprofilattico of Modena and Reggio Emilia, Reggio Emilia, Italy); all reagents necessary for in vitro studies, such as trypsin/EDTA, culture medium (DMEM) and fetal bovine serum (FBS) were obtained from GIBCO (Invitrogen Corporation, Paisley, UK). Finally, double distilled water was used to prepare all the samples.

### 4.2. Methods

#### 4.2.1. Liquid Crystal Nanosystems and Gelled LCNs Preparation

Three batches for each formulation (Table 1) were prepared following the protocol previously described with slight modifications [12]. Briefly, the organic phase was prepared putting GMO and the other lipid components (DSPE-PEG 2000 or DSPE-PEG 750) in a Pyrex^®^ glass vial and dissolved in chloroform/ethanol mixture (5 mL—10:90 *v*/*v*). Poloxamer P407 was dissolved in water (10 mL) in a second glass vial. To facilitate the solubilization of poloxamer P407, the aqueous phase was maintained for 1 h at 4 °C using magnetic stirring. The organic phase was added dropwise to the aqueous phase while continuously stirring (10,000 rpm) using the Ultraturrax T25 homogenizer (IKA^®^—Werke GmbH & Co., KG, Staufen, Germany). Once the organic phase has been added, ten homogenization cycles (thirty-seconds each) at 15,000 rpm with 1 min rest between them were carried out. Finally, the formulation was maintained under continuous stirring (Orbital Shaker KS 130 Control, IKA-WERKE, Staufen, Germany) at room temperature until any trace of the organic solvent has been removed. For the preparation of RSV-loaded LCNs, the amount of drug (0.24, 0.48 and 0.72 mg/mL) was dissolved in organic phase during the preparation of LCNs.

#### 4.2.2. Preparation of Empty Gels and Gelled LCNs

Empty gels were prepared via simply mixing gelling agents P407 20% (*w*/*w*) and CMC 5% (*w*/*w*) with the right amount of distilled water [46]. In the case of P407-based gels, the procedure was carried out at temperature of 4 °C to facilitate the dissolution of gelling agent. For the preparation of gelled nanosystems, LCNs were concentrated by using polycarbonate tubes and centrifuged (Avanti 30, Beckman, Fullerton, CA, USA) at 4 °C and 28,000 rpm. The filtered liquid was separated from semisolid mass of nanosystems blocked on the filter, and this last one was added to the gel formulation (2% *w*/*w*) for the subsequent studies [27].

#### 4.2.3. Physico-Chemical Characterization of LCNs by DLS and Turbiscan Lab^®^ Expert

Dynamic light scattering (DLS) was used to determine mean size, size distribution and zeta potential (Z-potential). For this purpose, a Zetasizer Nano ZS (Malvern Instruments, Worcestershire, UK), equipped with a laser diode having a rated output of 4.5 mV at a wavelength of 670 nm and a backscattering angle of 173 °C, was used. For the analysis, each sample was diluted with a suitable medium to avoid multi-scattering phenomena. The same tool was also used for the evaluation of the Z-potential of the vesicular suspensions by applying a Smoluchowsky constant F (Ka) of 1.5 as a function of the electrophoretic mobility of the samples. The various measurements were performed on three different batches (10 determinations for each batch). Results were expressed as the mean of three different experiments ± standard deviation.

For stability analysis, the Turbiscan Lab^®^ Expert was used by evaluating the variations in the delta-backscattering (ΔBS) and the delta-transmittance (ΔT) profiles of empty and RSV-loaded LCNs. The analysis was carried out at room temperature (25 °C) for 1 h. The kinetic stability profiles of the various samples were investigated and compared to each other by expressing the data as Turbiscan Stability Index (TSI) versus time (the lower the TSI, the greater the stability of the formulation) [37,47].

#### 4.2.4. Entrapment Efficiency of Resveratrol

The entrapment efficiency of resveratrol was evaluated by means of suitable spectrophotometric analysis. The purified RSV-loaded LCNs were centrifuged using Amicon^®^ Ultra centrifugal filters (4000 rpm for 60 min). The LCNs entrapped within filter was recovered, destroyed by using ethanol, and the entrapped amount of drug was spectrophotometrically determined (Lambda 35; Perkin Elmer, Waltham, MA, USA) at λ max 306 nm [48,49,50]. No interference deriving from the empty formulation was observed. The amount of drug entrapped in the LCNs was determined by using Equation (1) as described below:

(1) $$EE\;\left(\%\right)=\frac{De}{Dt}\times 100$$

where *De* is the amount of drug detected within centrifuged LCNs, and *Dt* is the amount of drug used for the LCNs preparation.

To be sure of the obtained results, a counter-test was carried out, analyzing the quantity of drug present in the liquid eluted during centrifugation of LCNs using Amicon^®^ Ultra centrifugal filters, and by using the following Equation:

(2) $$EE\;\left(\%\right)=\frac{Dt-Du}{Dt}\times 100$$

where *Dt* is the amount of drug used for the LCNs preparation, and *Du* is the amount of untrapped drug detected within eluted liquid.

Experiments were carried out in triplicate and the results were the average of three different experiments ± standard deviations.

In addition, the loading capacity (LC%) was calculated as the percentage of the ratio between the amount of total entrapped RSV and the total weight of the nanostructures.

#### 4.2.5. In Vitro Studies Carried out on NCTC 2544 Cells: Evaluation of Safety Profile and Improved Antioxidant Activity of RSV-Loaded LCNs

The human keratocytes NCTC 2544 cell line were incubated using suitable culture medium: DMEM medium added with penicillin (100 UI/mL), streptomycin (100 µg/mL), amphotericin B (250 µg/mL) and fetal bovine serum (10% *v*/*v*). When cellular confluence reached 80%, the trypsin was used to detached cells that were centrifuged and resuspended. For cytotoxicity evaluation, NCTC 2544 were placed in 96-well culture dishes at a density of 5000 cells/0.2 mL, while for antioxidant studies the cells were placed at a density of 8000 cells/0.2 mL.

The in vitro evaluation of LCNs safety profile was carried out treating NCTC 2544 for 24, 48 and 72 h with amounts of nanosystems equal to that necessary to deliver concentrations of RSV equal to 0.5, 1 and 5 µM. The study was carried out by using MTT test and the results were expressed in terms of cell viability (%), calculated as previously reported [51]:

(3) $$Cell\;Viability=\frac{Abs\;T}{Abs\;C}\times 100$$

Considering *Abs T* and *Abs C* the absorbance of treated and untreated (control) cells, respectively. For the quantification, the Varioskan™ LUX microplate reader (Thermo Fischer Scientific, Waltham, MA, USA) was used at λ = 540 nm and λ = 690 nm.

For the evaluation of protective effects of RSV-loaded LCNs, NCTC 2544 cells were previously treated for 24 h with 0.5 and 1 µM of RSV as free and entrapped form. After this prefixed time, cells were exposed to H_2_O_2_ (700 µM) for 1.5 h to induce oxidative stress. The protective effects of treatments were analyzing by using MTT test as described above and via lactic hydrogenase (LDH) assay. This last test is based on the LDH release obtained when an alteration or disruption of cell membrane occur, as in the case of H_2_O_2_ treatment. Pierce LDH cytotoxicity assay kit was used, and its specific protocol was followed. At the end of all treatments, LDH release was analyzed using a spectrophotometer in the cultured medium at λ = 680 nm and λ = 490 nm, and calculated as reported:

(4) $$\;LDH\;released\;\left(\%\right)=\frac{LDH\;compounds-LDH\;spontaneous}{LDH\;maximum-LDH\;spontaneous}\times 100$$

Equation (4) considers the absorbance of LDH released from treated cells (with H_2_O_2_ and with or without RSV) (*LDH compounds*), of LDH spontaneously released from untreated cells (*LDH spontaneous*) and of LDH recorded after lysis of cells (*LDH maximum*). The results were reported as the average of five different experiments ± standard deviation.

#### 4.2.6. Micro-Rheological Characterization of Empty Gels and Gelled LNCs through Diffusing Wave Spectroscopy (DWS)

Micro-rheological characterization of each sample was carried out by using the diffusing wave spectroscopy (DWS) (Rheolaser Master™, Formulaction). The analysis is based on the measurement of the particles’ Brownian motion which depends on the viscoelastic feature of our sample. For a better detection of microrheological behavior, each sample (20 mL) was added using latex beads (1 μm, 0.1 wt. %). During the time of analysis (1 h), the backscattered light intensity was detected, obtaining mean square displacement (MSD) as quantification of the sample particle movement as a function of time. Starting from MSD data, the RheoSoft Master 1.4.0.0. was able to provide other microrheological parameters, namely elasticity index (EI) and solid liquid balance (SLB).

#### 4.2.7. Dynamic Rheological Evaluation of Empty Gels and Gelled LNCs by Using Kinexus Pro+ Rotational Rheometer

Dynamic rheological characterization of all gelled samples was carried out by using a Kinexus Pro+ rotational rheometer (Malvern Panalytical Ltd., Spectris plc, Malvern WR14 1XZ, UK), equipped with cone-plate geometries (40 mm diameter; 2° angle). All runs were carried out at 25.00 ± 0.01 °C. A fixed gap between the geometries was pre-set to 1 mm and the excess sample was removed. The obtained rheological data was processed by the rSpace software v.2.1. Compressed air flow (2 bar), pre-filtered through fine and superfine Clearpoint filters (Beko, Atlanta, GA, USA), allowed for reaching the pressure able to perform the analysis. Before each analysis, the samples were carefully and gently loaded onto the lower measuring plate of the rheometer and the upper measuring geometry was lowered at very slow speed to prevent the alteration of sample structure. Preliminary tests were needed to define suitable experimental conditions for rheological measurements maintaining within the linear viscoelasticity region (LVR). For this purpose, sweep strain tests (0.01 to 100%) were carried out at a frequency equal to 1 Hz.

Viscosity studies were performed by means of a shear rate ramp starting from 0.1 s^−1^ and ending to 100 s^−1^. Other analyses were carried out to characterize all gelled samples by means oscillatory test with a frequency sweep ranging from 0.1 Hz to 10 Hz and at controlled stress (1 Pa), obtaining important information about storage modulus G′ (elastic component) and loss modulus G″ (viscous component).

#### 4.2.8. In Vitro Percutaneous Permeation Profiles of RSV from LCNs and Gelled LCNs

The in vitro percutaneous permeation studies were carried out by using dynamic diffusion Franz cells and following a previous consolidated method. The diffusional surface area and the receptor volume of diffusion cell are equal to 0.75 cm^2^ and 4.75 mL, respectively. To mimic the interaction of nanosystems and human skin, the studies were carried out interposing stratum corneum and viable epidermis (SCE) membrane between donor and receptor compartments of Franz cells. The tissue was obtained from human skin following abdominal reduction surgery, and it was isolated as previously described [52]. Before starting the investigation, the integrity of human SCE membranes was checked by evaluating the transepithelial electrical resistance (TEER) [53].

Considering the lipophilicity of RSV, the receptor chamber was filled with a hydro-alcoholic solution (H_2_O:EtOH 70:30 *v*/*v*) to guarantee sink conditions, and the medium was constantly stirred and warmed to 37 ± 1 °C. In donor receptors, 200 µL of each formulation were loaded then the donor compartment was sealed by using Parafilm^®^ to induce occlusive conditions. The percutaneous permeation was monitored for 8 h, and at prefixed times, 1 mL of receptor fluid was withdrawn and analyzed using a UV-Vis spectrophotometer. Each aliquot of receptor fluid was replaced with fresh fluid. Three different experiments were carried out and the results were expressed as mean values ± standard deviation.

#### 4.2.9. Evaluation of Safety Profile and Skin Tolerability Evaluation of LCNs and Gelled LCNs on Healthy Human Volunteers

The skin tolerability and the consequent skin safety profile of proposed gelled formulations were evaluated on healthy human volunteers by monitoring the variation of transepidermal water loss (TEWL) using the C + K Multi Probe Adapter equipped with Tewameter^®^ TM300 probe (Courage & Khazaka, Cologne, Germany) [54]. This apparatus allows the indirect measurement of water evaporation gradient through the skin. for the experiments, ten healthy human volunteers (both sexes with an average age of 26 ± 2 years) have been fully informed regarding the nature and the potential risks of the study and they signed an informed consent form, approving the experimental protocol.

Before starting the in vivo evaluation, the volunteers were accommodated for 1 h in a day surgery room at 24 ± 1 °C and 40–50% r.h.

Six sites (one for each sample: saline solution as negative control, empty occlusive patch as positive control, 5% CMC-based gels with LCN-A, 5% CMC-based gels with LCN-C, 20% P407-based gels with LCN-A and 20% P407-based gels with LCN-C) were marked on the ventral surface of each subject’s forearm, at least 2 cm of distance between each site. Occlusive commercial patches (Farmacosmo S.r.l., Napoli, Italy) were used to allow the application of 150 μL of each sample. At fixed times (1, 2, 4, 6 and 8 h), the patches were gently removed from the skin and data (TEWL g/h·m^2^) was collected by Tewameter^®^ TM300 and processed by Courage & Khazaka software MPA.

All in vivo studies were carried out in accordance with the Declaration of Helsinki, and the protocol was approved by the Research Ethics Committee of the University of Catanzaro “Magna Græcia” (Approval number: 392/2019).

#### 4.2.10. Statistical Analysis

One-way ANOVA was used for statistical analysis of obtained data. A Bonferroni *t*-test was used to validate the results, defining * *p* ≤ 0.05 and ** *p* ≤ 0.001 as statistically significant. All values are reported as average ± standard deviation.

## Figures and Tables

**Table 1:** Chemical composition of different LCNs formulation.

Formulation	GMO (mg)	P407 (mg)	DSPE-PEG 2000 (mg)	DSPE-PEG 750 (mg)
LNC-A	90	50	-	-
LNC-B	90	50	3	-
LNC-C	90	50	-	3

**Table 2:** Physico-chemical features and entrapment efficiency of LCNs containing three different concentrations of RSV. The data are expressed as mean value ± standard deviation. The various measurements were carried out in triplicate on three different batches.

Formulation	Concentration (mg/mL)	Mean Size (nm)	PdI	Z-Potential (mV)	EE (%)	LC (%)
LCN-A	-	96 ± 0	0.13 ± 0.02	−22 ± 1	-	-
RSV-A 0.24	0.24	99 ± 1	0.18 ± 0.05	−23 ± 1	19 ± 1	0.32 ± 0.01
RSV-A 0.48	0.48	98 ± 0	0.10 ± 0.00	−25 ± 2	39 ± 2	1.33 ± 0.03
RSV-A 0.72	0.72	95 ± 2	0.11 ± 0.01	−19 ± 1	49 ± 1	2.52 ± 0.02
LCN-B	-	82 ± 0	0.17 ± 0.01	−34 ± 1	-	-
RSV-B 0.24	0.24	83 ± 1	0.15 ± 0.01	−35 ± 4	21 ± 2	0.35 ± 0.00
RSV-B 0.48	0.48	81 ± 0	0.19 ± 0.01	−39 ± 1	22 ± 3	0.74 ± 0.02
RSV-B 0.72	0.72	83 ± 3	0.22 ± 0.01	−31 ± 1	18 ± 2	0.90 ± 0.01
LCN-C	-	79 ± 1	0.19 ± 0.01	−28 ± 2	-	-
RSV-C 0.24	0.24	73 ± 1	0.22 ± 0.00	−36 ± 6	26 ± 1	0.44 ± 0.03
RSV-C 0.48	0.48	82 ± 1	0.24 ± 0.01	−36 ± 1	36 ± 1	1.21 ± 0.01
RSV-C 0.72	0.72	84 ± 2	0.23 ± 0.00	−34 ± 1	39 ± 2	1.96 ± 0.00

**Table 3:** Shear viscosity (Pa·s) values recorded at specific shear rate (**γ**) for gelled samples at 25 °C. Values are reported as the average of three independent experiments ± standard deviation.

	Shear Viscosity (ɳ—Pa·s)			
Formulation	γ = 0.1 s^−1^	γ = 1 s^−1^	γ = 10 s^−1^	γ = 100 s^−1^
5% CMC	10.6 ± 2.1	9.25 ± 0.96	6.33 ± 1.11	2.80 ± 0.89
5% CMC-LCN-A	7.9 ± 1.5	6.70 ± 1.04	4.77 ± 0.79	2.07 ± 0.69
5% CMC-LCN-C	4.1 ± 1.6	3.44 ± 0.63	2.75 ± 0.54	1.29 ± 0.54
20% P407	964.70 ± 10.23	232.40 ± 8.52	25.87 ± 5.21	3.35 ± 0.78
20% P407-LCN-A	818.50 ± 13.02	164.20 ± 10.24	19.62 ± 2.45	2.67 ± 0.63
20% P407-LCN-C	1003.00 ± 21.03	217.90 ± 7.12	24.38 ± 2.06	3.10 ± 0.21

## Data Availability

The data presented in this study are available from the corresponding author upon request. The data are not publicly available due to privacy.
